# Socioeconomic influence on treatment with liothyronine and desiccated thyroid extract in Denmark

**DOI:** 10.1530/ETJ-22-0149

**Published:** 2022-09-27

**Authors:** Jeppe Lerche la Cour, Line Tang Møllehave, Bjarke Røssner Medici, Christian Zinck Jensen, Anne Ahrendt Bjerregaard, Birte Nygaard

**Affiliations:** 1 Center for Endocrinology and Metabolism, Department of Internal Medicine, Herlev and Gentofte Hospitals, Herlev, Denmark; 2 Center for Clinical Research and Prevention, Bispebjerg and Frederiksberg Hospital, Capital Region, Denmark; 3 Faculty of Health and Medical Sciences, University of Copenhagen, Copenhagen, Denmark

**Keywords:** liothyronine (T3), socioeconomic, desiccated thyroid extract (DTE), social inequality, hypothyroidism, incidence

## Abstract

**Introduction:**

High compared with low educational level increases the odds of starting levothyroxine (L-T4) with a normal thyroid-stimulating hormone – the mechanism is most likely patient request. The use of liothyronine (L-T3) and desiccated thyroid extract (DTE) is also speculated to be initiated at patients’ request. Therefore, the primary aim of this study was to evaluate if educational level influences treatment with L-T3 and DTE.

**Material and methods:**

In this register-based cross-sectional study, we included all Danish citizens ≥30 years with redeemed prescription of L-T4, L-T3, or DTE during 2017–2020. We defined educational levels as short, medium, and long (<10 years, 10–12 years, and above 12 years, respectively). The association between educational level and treatment with LT3 or DTE vs only LT4 was analyzed in logistic regression models adjusted for age and sex.

**Results:**

We included 154,360 individuals using thyroid medication of whom 3829 were treated with L-T3 (2.48%) and 430 with DTE (0.28%). The usage was highest among women (3.15%) and the age group 40–49 (5.6%). Longer education compared with short increased the odds of being treated with DTE or L-T3 (medium education odds ratio (OR) 1.61 (95% CI 1.50–1.8) and long education OR 1.95 (95% CI 1.79–2.13)). Test for trend: OR: 1.37 (95% CI 1.31–1.42). Adjustment for other covariates did not affect the results substantially.

**Conclusion:**

Persons with a longer compared to a shorter education are more often treated with either DTE or L-T3, and the usage of these drugs is limited to less than 3% of thyroid hormone users.

## Introduction

Persons treated with levothyroxine (L-T4) describe a decreased quality of life more often than the general population and the explanation seems to be multifactorial. Whether to use routine treatment of hypothyroidism to increase the quality of life has been debated for many years (1), while the use of thyroid hormone has increased worldwide. In Denmark, from 2001 to 2015, an increase of 132% was seen, simultaneously, with a decrease in the thyroid-stimulating hormone (TSH) threshold for initiating L-T4 therapy and approximately 25% started treatment without biochemical hypothyroidism (2). The increase in L-T4 therapy and falling threshold may be due to increasing demand from patients (3). A recent study demonstrates that the probability of starting thyroid hormone therapy despite normal TSH was higher in persons with long education (4). Educational level is a marker of social status and may indicate the ability to access health information and increased argumentative skills (5). Thus, we speculate that these patients were more likely to request off-label treatments in a dialog with the general practitioner (GP).

During the last few decades, the interest in thyroid hormone treatments – not ‘just L-T4’ – has increased, and it has been suggested that some hypothyroid patients could improve their quality of life if treated with liothyronine (L-T3) or desiccated thyroid extract (DTE). However, L-T3 and DTE therapy is still controversial among endocrinologists and GPs, due to the lack of evidence of treatment effect in large-scale randomized clinical trials. Guidelines suggest that L-T4 is standard therapy. If symptoms persist after normalization of TSH, combination therapy with L-T3 may be considered an experimental approach according to the European guidelines (ETA) (1) but not the American guidelines (ATA) (6). Testing and prescribing on patients’ request is known to happen (3) and people with longer education more often seek the medical system (5). Consequently, we speculated that educational level impacts the usage of L-T3 and/or DTE as seen in treatment initiation for L-T4 with normal TSH.

### Aim

The primary aim was to evaluate if educational level influenced treatment with L-T3 and DTE prescribed by Danish doctors.

Further, we wanted to address influences from age, sex, income, region of residence, depression, and prior treatment with thyroid surgery or radioiodine.

## Methods

### Study population

Registries at Statistics Denmark contain unique individual-level data from nationwide administrative registers on educational level, income, age, sex, region of residence, and treatments and are updated annually. The Danish National Prescription registry contains records of all redeemed prescriptions since 1995 (7) and L-T3 and DTE since 2017.

We included all citizens in Denmark ≥30 years with at least one redeemed prescription of the thyroid hormones L-T4, L-T3, or DTE ((ATC: H03AA), including L-T4 H03AA01, L-T3 H03AA02, and DTE H03AA05) during 2017–2020.

We excluded patients with a diagnosis that may be a contraindication to L-T3 treatment: atrial fibrillation (ICD10: DI49, DI48, DI47 except for DI471E+F), a redeemed prescription of amiodarone (ATC: C01BD01) ever, thyroid cancer (ICD-10: DC73.9) ever, and those who redeemed a prescription of antithyroid medication 2 years before redeeming T3 and/or DTE to exclude current block/replace treatment. Further, we chose a grace period with pregnancy or abortion ± 1 year.

According to Danish legislation, ethical approval is not required for studies based solely on registers. We obtained approval for handling the data from the Danish Data Protection Agency, Statistics Denmark, and the Health Data Authorities. All analyses were performed on servers at Statistics Denmark with pseudonymized personal identification numbers.

### Exposure and outcomes

The highest attained educational level was the primary exposure. Educational level was classified as short (primary or upper secondary education), medium 10–12 years, and long (≥12 years: bachelor’s or master’s degree or short-cycle higher education). Educational level was defined in the year of the latest redeemed prescription.

The primary outcome was one or more redeemed prescriptions of L-T3 or DTE vs only L-T4 during 2017–2020.

### Covariates

We defined age, sex, and region of residence in the year of the latest redeemed prescription. The disposable income quartile was defined for each subject as the equivalized disposable income (total disposable household income divided by the Organisation for Economic Co-operation and Development equivalent size of the household) and classified by the disposable income quartiles for the entire adult Danish population each calendar year.

Thyroid surgery (procedure codes: BAA20-BAA60(A)) and radioiodine (procedure codes BWGGI and WT(F-L)RNJLXX) treatment performed after 1990 and 2004, respectively, were registered. Finally, anti-depressive medication (ATC group N06A) (excluding Zyban/bupropion (N06AX12) and Duloxetine (N06AX21) because they are also used for antismoking and for overactive bladder) redeemed after 1995 were registered.

### Statistical analyses

Population characteristics are presented for the total population and stratified by type of hypothyroid medication redeemed. Logistic regression models were applied to assess the odds ratios (OR) and 95% CI of the associations between educational level and L-T3 or DTE vs only L-T4 and the associations between further patient characteristics (age, sex, income, region of residence, depression, and prior treatment with thyroid surgery or radioiodine) and L-T3 or DTE vs only L-T4. We adjusted the analyses for potential confounding variables in several models, including age, sex (model 1), treatments including thyroid surgery, radioiodine, antidepressant medication (model 2), region of residence, and income (model 3), and finally, model 4 comprising all covariates of model 1 through 3 except income in the primary analyses with education as exposure. As models 2 and 3 are interim, data from these are not shown. Age and sex were included in all models. Dose–response relationship was assessed by testing the linear effect of the ordinal variable.

In sensitivity analyses, the effect of educational level as a two-classed variable was examined. Disposable income was included in sensitivity analyses; although income heavily depends on education, the two measures are not the same (8).

Analyses were performed at servers at Statistics Denmark in SAS, version 9.4 (SAS Institute Inc, Cary, NC, USA), and statistical significance was defined as *P*  <0.05.

## Results

**Figure 1:**
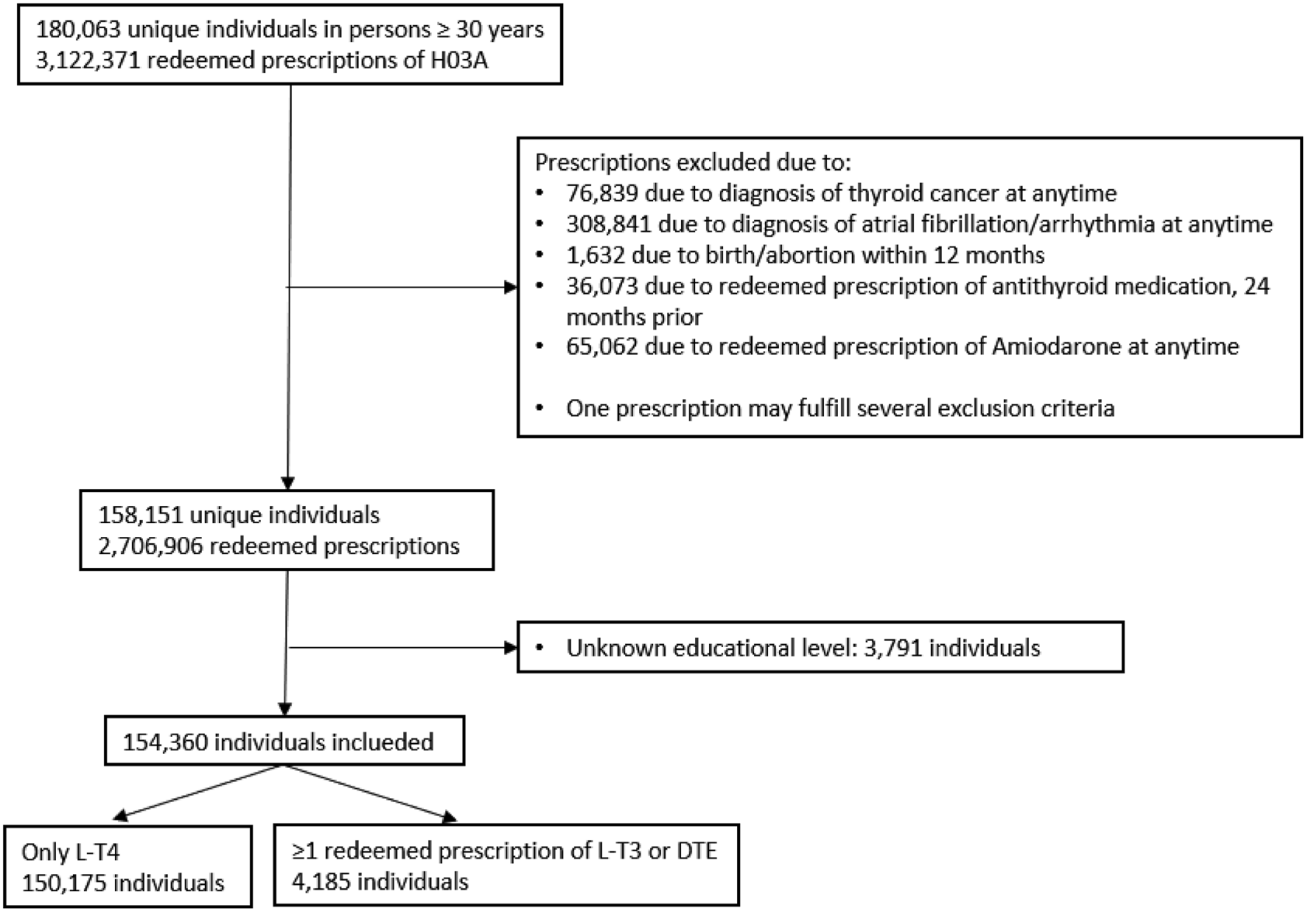
Consort diagram.

We identified 180,063 persons who redeemed thyroid hormone replacement prescriptions and ended up including 154,360 persons in the study (see Fig. 1). In short, of the total study population, 3829 (2.48%) were treated with L-T3 and 430 (0.28%) with DTE. The majority taking L-T3 and/or DTE were women (97.3%), and the most common age for this was 40–59 years (57.9%). For further description, see Table 1.

**Table 1:** Characteristics of patients treated with thyroid hormones.

Variable	All (*n*)	Mono therapy L-T4 (% (*n*))^a^	L-T3 (% (*n*))^b^	DTE (% (*n*))^c^
Total	154,360	97.29 (150,175)	2.48 (3829)	0.28 (430)
Age				
30–39	14,236	97.69 (13,907)	2.21 (315)	0.13 (19)
40–49	20,949	94.51 (19,799)	5.06 (1060)	0.55 (116)
50–59	30,530	95.20 (29,065)	4.42 (1350)	0.46 (140)
60–69	33,775	97.46 (32,916)	2.28 (769)	0.30 (103)
70+	54,870	99.30 (54,488)	0.61 (335)	0.09 (52)
Sex				
Men	24,742	99.24 (24,555)	0.73 (180)	0.03 (8)
Women	129,618	96.92 (125,620)	2.82 (3649)	0.33 (422)
Educational level				
Short, % (*n*)	51,142	98.51 (50,379)	1.33 (681)	0.19 (96)
Medium, % (*n*)	53,293	97.34 (51,876)	2.44 (1299)	0.28 (148)
Long, % (*n*)	49,925	95.98 (47,920)	3.70 (1849)	0.37 (186)
Disposable income^d^				
First quartile, % (*n*)	46,024	98.61 (45,383)	1.22 (561)	0.19 (89)
Second quartile, % (*n*)	41,915	97.75 (40,970)	2.04 (855)	0.25 (105)
Third quartile, % (*n*)	33,449	96.51 (32,280)	3.21 (1074)	0.34 (115)
Fourth quartile, % (*n*)	32,456	95.63 (31,038)	4.09 (1329)	0.37 (119)
Medication against depression				
No, % (*n*)	93,582	97.57 (91,306)	2.24 (2100)	0.23 (213)
Yes, % (*n*)	60,778	96.86 (58,869)	2.84 (1729)	0.36 (217)
Thyroid surgery				
No, % (*n*)	141,967	97.32 (138,164)	2.45 (3473)	0.28 (395)
Yes, % (*n*)	1393	96.92 (12,011)	2.87 (356)	0.28 (35)
Radioiodine				
No, % (*n*)	149,308	97.29 (145,263)	2.48 (3699)	0.28 (420)
Yes, % (*n*)	5052	97.23 (4912)	2.57 (130)	0.20 (10)
Region of residence				
Capital, % (*n*)	48,421	96.38 (46,667)	3.30 (1600)	0.38 (186)
Zealand, % (*n*)	26,987	96.95 (26,165)	2.69 (727)	0.43 (116)
South, % (*n*)	32,706	98.15 (32,102)	1.68 (548)	0.19 (61)
Midt, % (*n*)	32,559	98.11 (31,944)	1.79 (583)	0.11 (37)
North, % (n)	13,687	97.15 (13,297)	2.71 (371)	0.22 (30)

^a^Monotherapy L-T4 only received L-T4 during the study period; ^b^Patients receiving T3 can also have received L-T4 and/or DTE during the study period; ^c^Patients receiving DTE can also have received L-T4 and/or T3 during the study period; ^d^Disposable income quartiles of the whole adult Danish population.

Results of logistic regression models 1 and 4 are presented in Table 2. When analyzing whether educational level influenced treatment with L-T3 and DTE, we found that the odds rose with an increasing educational level for medium vs short 1.64 (95% CI: 1.50–1.8) and long vs short 1.95 (95% CI: 1.79–2.13) in the age- and sex-adjusted model. Test for trend: OR: 1.39 (95% CI: 1.33–1.44) *P* < 0.001) (Table 2). The results only changed marginally in the partly adjusted models (models 2+3) and in sensitivity analyses including disposable income as a covariable (results not shown). The same pattern of increasing odds of treatment with L-T3 or DTE was seen when increasing disposable income was applied as the exposure in the sensitivity analysis.

**Table 2:** Influence of population characteristics on odds of being treated with liothyronine (L-T3) or desiccated thyroid extract (DTE).

Variable	*n* LT3 or DTE/*n*	Sex- and age-adjusted model, OR (95% CI)	Fully adjusted model^a^, OR (95% CI)
Age			
30–39	329/14,236	1 (Ref)	1 (Ref)
40–49	1150/20,949	2.53 (2.23–2.87)	2.51 (2.21–2.84)
50–59	1465/30,530	2.22 (1.96–2.50)	2.18 (1.93–2.47)
60–69	859/33,775	1.15 (1.02–1.31)	1.21 (1.06–1.38)
70–79	324/32,649	0.45 (0.38–0.52)	0.54 (0.46–0.63)
80+	58/22,221	0.12 (0.09–0.15)	0.16 (0.12–0.21)
Sex			
Men	187/24,742	1 (Ref)	1 (Ref)
Women	3998/129,618	3.96 (3.42–4.59)	3.73 (3.22–4.33)
Educational level			
Short	763/51,142	1 (Ref)	1 (Ref)
Medium	1417/53,293	1.64 (1.50–1.80)	1.68 (1.53–1.83)
Long	2005/49,925	1.95 (1.79–2.13)	2.01 (1.84–2.20)
Disposable income			
First quartile	641/46,024	1 (Ref)	1 (Ref)
Second quartile	945/41,915	1.49 (1.35–1.65)	1.45 (1.31–1.61)
Third quartile	1169/33,449	2.01 (1.82–2.22)	1.93 (1.74–2.14)
Fourth quartile	1418/32,456	2.54 (2.31–2.79)	2.38 (2.15–2.64)
Medication against depression			
No	2276/93,582	1 (Ref)	1 (Ref)
Yes	1909/60,778	1.37 (1.28–1.45)	1.65 (1.55–1.76)
Thyroid surgery			
No	3803/141,967	1 (Ref)	1 (Ref)
Yes	382/12,393	1.18 (1.06–1.31)	1.20 (1.08–1.34)
Radioiodine			
No	4045/149,308	1 (Ref)	1 (Ref)
Yes	140/5052	1.10 (0.93–1.31)	1.21 (1.02–1.44)
Region of residence			
Capital	1754/48,421	1 (Ref)	1 (Ref)
Zealand	822/26,987	0.94 (0.86–1.02)	0.96 (0.88–1.05)
South	604/32,706	0.55 (0.50–0.61)	0.57 (0.52–-0.63)
Middle	615/32,559	0.55 (0.50–0.61)	0.55 (0.50–0.61)
North	390/13,687	0.90 (0.80–1.00)	0.95 (0.85–1.06)

^a^Adjusted for age, sex, treatments (thyroid surgery, radioiodine, and depression medication), region of residence, and educational level. Income is not included in the model for education because this is considered a clear mediator.

Further, we found an age-dependent probability of treatment with L-T3 and/or DTE with age 40–49 years at highest odds compared with age 30–39 (OR 2.53 (95% CI: 2.23–2.87)) decreasing towards lower odds with age ending at age 80+ (OR 0.12 (95% CI: 0.09–0.15)) (Table 2). We found a higher OR of a redeemed prescription of DTE or L-T3 after thyroid surgery OR 1.18 (95% CI: 1.06–1.31) and medication for depression OR 1.37 (95% CI: 1.28–1.45). Also, the region of residence played a role as residents in the Capital region had higher ORs of treatment with DTE or L-T3 than residents in the Middle or South regions (Table 2). As for educational level, the results of all other exposures only changed marginally in the adjusted models.

## Discussion

In this Danish national register-based cross-sectional study, we found that less than 3% of persons treated with thyroid hormones were treated with either L-T3 or DTE from 2017 to 2020. Compared with short education, there is almost double the odds (OR 1.95) that people with a long education are treated with L-T3 or DTE, illustrating social inequality in treatment. Further, we find that sex, age, former thyroid surgery, region of residence, and medical therapy with antidepressants played a role in the odds of treatment but only a minor role compared to education level.

Treatment with L-T3 or DTE is still controversial. Current guidelines from ETA and ATA are different on the issue of L-T3 supplementary treatment, where ETA is open for a trial period – ATA advises against routine use due to a lack of evidence of benefit (1, 6). Both agree upon not recommending DTE. At the same time, there is a consensus statement between ETA, the British thyroid association, and ATA that more knowledge is needed on the effectiveness and side effects of combination therapy (9); thus, the debate on whether to use L-T3 is ongoing. The discussion on DTE, which most endocrinologists consider obsolete, might be refueled by the recent blinded crossover study on DTE vs L-T4 and L-T3 with 75 patients (10). In this study, the overall result was neutral, but they postulate that the patients having the lowest quality of life had an effect of L-T3/DTE from a subgroup analysis. Currently, there are more neutral/negative trials on LT4 supplementary treatment (DTE and L-T3), but still, many questions are unanswered as stated in the formerly mentioned consensus statement (9).

Initiation of therapy, not recommended as standard treatment in Guidelines, will often be based on a combination of patient requests and the willingness of the doctors to initiate a specific therapy.

Regarding the doctor’s willingness to initiate combination therapy, a recent international questionnaire (THESIS study) asked endocrinologists if they would prescribe L-T3 combination therapy in L-T4-treated hypothyroid patients with persistent symptoms. In Denmark, 71% answered yes (11), which is like Swedish data (78%) (12) but is much higher compared with other European countries, i.e. Italy (40%) and France (26%) (13). The difference in doctors’ willingness could be explained by local guidelines and the availability of the medication. The patient request is related to the national focus of patients’ societies and social media on the possible effects of this specific therapy. In Denmark, high focus and availability of the medication are present, and the Danish Endocrine Society guidelines suggest a trial of L-T3 therapy (but not DTE) in patients with persistent symptoms despite long-term euthyroidism on L-T4.

Sometimes patients persuade the doctors to prescribe other treatments than the treatments the doctors prefer according to guidelines. The known discrepancy between patients’ wish for treatment and doctors wanting to follow guidelines is supported by a qualitative study from the USA among ATA members showing that patients’ wish was reported as a barrier to therapy following guidelines (14). The same mechanism may be the case with L-T3 treatment.

If we believe in a possible effect of L-T3/DTE, the number of patients treated with these drugs is low (<3%) – compared with the estimate that 5–10% may be undertreated with L-T4 alone (15, 16). On the other hand – if the treatment is harmful, many patients are harmed unnecessarily. We lack solid randomized sufficiently powered long-term trials to know whether side effects do exist. There are, however, newer retrospective data that raises concerns with one study showing increased risk of heart failure and possible stroke and another with increased risk of anti-psychotic prescription (17, 18).

It is not a surprise that educational level and sex influence prescriptions of DTE and L-T3, as it was recently described that educational level (as an indicator of cognitive and analytical skills (5)) was a factor in initiating L-T4 treatment with normal TSH (4). The same applies to the fact that women are more prone to DTE and L-T3 therapy and starting therapy with normal TSH (19). We speculate that a similar mechanism exists here.

We found that former thyroid surgery was related to initiating L-T3/DTE. We excluded those who had a total thyroidectomy due to thyroid cancer. By this, we excluded the often-used L-T3 pretreatment before radioiodine ablation or uptake measurement as the explanation. The explanation could be that this group of people are more often seen by endocrinologists who are more prone to start L-T3 treatment than GPs.

We also demonstrated that the region of residence was related to initiating this treatment. The explanation could be that local doctors’ preferences follow key opinion leaders that are more willing to start this treatment and vice versa. Another explanation could be that in some regions more people are getting DTE and/or L-T3 via the internet and are thereby not included in the database. In a previous study done by questionnaire, we estimated that about 5% of the patients in Denmark on L-T3/DTE buy the hormones on the internet (20), meaning that we most likely have data on 95% of the patients.

### Limitations

Many limitations mentioned were relevant in the article. Adding to these, we are limited by our study design being retrospective and based on registers. As all registered-based studies, we, unfortunately, cannot extract clinical data on BMI, smoking patients feeling of well-being, and other things that are not registered. Worse is that we are not able to investigate whether the diagnosis of hypothyroidism was correct. Further, we are not able to dissect whether it is the doctors in high educational regions that are more prone to prescribe L-T3 or DTE or the patients that request it. This is of course a limitation, and we cannot know with certainty if this is what drives our findings. Given our findings of regional differences, it may very well be doctors driving it as many regions are comparable in social economic data in Denmark.

## Conclusion

In this large epidemiological study on more than 154,000 persons treated with thyroid hormones, we discover that the odds of being treated with L-T3 or DTE almost doubled with the longest educational level compared with the shorter and that approximately 3% of persons treated with thyroid hormones were treated with L-T3 or DTE.

## Declaration of interest

The authors declare that there is no conflict of interest that could be perceived as prejudicing the impartiality of the research reported.

## Funding

This study received funding from the non-profit Musikforlæggerne Agnes og Knut Mørks Fond.

## Author contribution statement

J L C conceived the study and drafted the manuscript, L T M conceived the study and did the data analytical work and helped on the manuscript, B R M helped to conceive the study and helped on the manuscript, C Z J helped to conceive the study and helped on the manuscript, A A B helped to with the data analytical work and helped on the manuscript, B N conceived the study and helped on the manuscript.
